# Intervals as musical fingerprints: perceptual and pedagogical insights—a scoping review

**DOI:** 10.3389/fpsyg.2026.1740840

**Published:** 2026-02-16

**Authors:** Mustafa Kendüzler

**Affiliations:** Department of Music Education, Gazi University, Ankara, Türkiye

**Keywords:** auditory perception, interval training, music pedagogy, pitch cognition, solfege

## Abstract

The perception and training of musical intervals form a central foundation for pitch cognition and musical understanding within solfege education. This scoping review maps the existing literature on interval training, emphasizing the interplay between perceptual mechanisms, pedagogical modeling, and instructional design. Following the PRISMA-ScR guidelines, systematic searches were conducted across three academic platforms—ERIC, Google Scholar, and JSTOR—using combinations of the keywords “interval training,” “solfege,” “pitch perception,” and “music education.” Thirty-five peer-reviewed studies published between 2010 and 2025 were analyzed, while historical and theoretical sources (1889–2009) were also considered to contextualize the pedagogical and cognitive development of interval training. Thematic synthesis identified three overarching domains: (1) perceptual-cognitive mechanisms underlying interval recognition, tonal awareness, and auditory feedback; (2) pedagogical models integrating auditory, visual, and kinesthetic modalities in solfege instruction; and (3) instructional frameworks promoting pitch stability, long-term learning, and vocal-motor integration. Collectively, the reviewed evidence suggests that structured interval training enhances pitch discrimination, strengthens the mapping between perception and vocal production, and provides a sustainable foundation for music learning. By linking perceptual cognition with pedagogical practice, this study contributes to a broader understanding of how interval-based training supports both the psychological and educational dimensions of musical development.

## 1 Introduction

Pitch perception lies at the heart of human musical experience, governing how listeners organize, remember, and anticipate sound. Within this auditory landscape, intervals—the frequency relationships between successive tones—serve as the fundamental *“musical fingerprints”* that define melodic identity (Thompson, 2013, as cited in Wong et al., 2021, p. 1028). Each interval embodies a distinctive ratio between frequencies, and together these relations form the structural and emotional fabric of melody. As Levitin (2006) notes, interval recognition underpins the auditory system’s capacity to encode proportional pitch differences and maintain melodic constancy across transpositions. Because melody perception is relational rather than absolute, understanding intervals is central not only to cognitive theories of tonal organization but also to the acquisition of musicianship skills. In this context, Ferrer et al. (2014, p. 41) argue that the auditory identification of intervals and the determination of their qualitative characteristics constitute fundamental skills for all students pursuing music education.

Despite their perceptual centrality, intervals are often treated in music education as mechanical or theoretical abstractions—entities to be memorized rather than experienced as perceptual relationships. In solfege pedagogy, however, the goal extends beyond naming interval types to internalizing how they *sound* and *feel* in relation to a tonal center. Therefore, solfege education supports not only the development of technical skills but also the processes of musical meaning-making and creative expression (Kendüzler, 2025 p. 496). This transformation—from conceptual labeling to embodied perception—requires the integration of auditory, cognitive, and motor processes. Yet, empirical evidence connecting these perceptual foundations with solfege instruction remains fragmented. Most studies investigate interval perception in laboratory contexts, while pedagogical research emphasizes skill development without systematically addressing the underlying perceptual mechanisms. In contrast, recent studies suggest that interactive and inclusive techniques can enhance students’ pitch, interval, and melody recognition skills (Yuexiao and Meeson, 2024, p. 638). Bridging this divide is essential for aligning cognitive theory with applied music training.

A growing body of literature highlights interval training as a cornerstone of musical expertise. Dowling and Harwood, (1986, as cited in Zarate et al., 2012); demonstrated that melodic recognition depends on the retention of intervallic relationships rather than absolute pitches. Similarly, McDermott et al. (2010) and Crowder, (1984, as cited in Thompson, 2013) showed that the auditory system encodes interval magnitudes as relational constructs that remain stable under transposition, explaining why melodies are recognized across keys. Pedagogically, these perceptual principles manifest in solfege practice, where learners cultivate sensitivity to pitch distances through listening, vocalization, and kinesthetic feedback. However, instructional approaches to interval training vary widely—ranging from fixed-do solmization to movable-do systems, from reference-song associations to visual-kinesthetic hand signs—reflecting an absence of unified pedagogical synthesis. Understanding how perceptual and educational dimensions interact thus holds critical implications for both teaching and research.

Although interval training has long been embedded in solfege curricula, its theoretical grounding in perceptual science has rarely been systematically reviewed. Existing studies tend to isolate cognitive, pedagogical, or physiological dimensions, leaving their intersections underexplored. Moreover, there is limited integration between experimental findings on auditory cognition and applied strategies for teaching pitch accuracy, intonation, and tonal awareness. Addressing this gap requires a comprehensive synthesis that situates interval training at the nexus of perception, learning, and performance—thereby connecting the psychology of music with its educational practice.

The present study therefore conducted a scoping review to map the perceptual foundations and pedagogical implications of interval-based training within solfege education. Guided by the PRISMA-ScR framework, it synthesizes empirical and theoretical research spanning perceptual-cognitive mechanisms, instructional modeling, and innovative teaching frameworks. By linking auditory science with pedagogical application, this review aims to clarify how interval perception functions as both a psychological process and an educational tool. Ultimately, it seeks to establish an integrative conceptual framework through which solfege education can foster not only accurate intonation but also enduring perceptual awareness and expressive musicianship.

## 2 Methodology

### 2.1 Review design

This study adopted a scoping review approach in accordance with the PRISMA-ScR (Preferred Reporting Items for Systematic Reviews and Meta-Analyses Extension for Scoping Reviews) guidelines developed by Tricco et al. (2018).

A scoping design was chosen because the aim was not to statistically aggregate findings but to map, categorize, and conceptualize the scope, nature, and theoretical orientations of research addressing interval training within solfege education.

Following the PRISMA-ScR framework ensured methodological transparency, reproducibility, and breadth, particularly in defining research questions, search parameters, inclusion criteria, and thematic synthesis procedures. This design also enabled the integration of historical, theoretical, and empirical sources, making it suitable for a multidimensional field where perceptual, cognitive, and pedagogical perspectives converge.

### 2.2 Search strategy

Comprehensive searches were conducted across three major academic platforms—ERIC, Google Scholar, and JSTOR—covering publications from 2010 to 2025.

The following Boolean combinations were used:

(“interval training” OR “interval perception”) AND (“solfege” OR “solfège”) AND (“pitch perception” OR “intonation” OR “music education”).

Reference lists of all eligible studies were manually screened to identify additional sources. The search was restricted to peer-reviewed journal articles, books, book chapters, and conference proceedings written in English or Turkish. Selected dissertations and historical treatises were also included to contextualize the conceptual and pedagogical evolution of interval theory.

### 2.3 Inclusion and exclusion criteria

Studies were included if they:

(a) addressed interval perception, solfege instruction, auditory cognition, pitch accuracy, or pedagogical approaches in music education; and
(b) provided either empirical evidence or theoretical discussion relevant to perceptual or pedagogical dimensions of interval training.

Studies were excluded if they:

(a) focused solely on instrumental tuning or acoustical physics;
(b) presented neurological or computational findings without educational interpretation; or
(c) lacked reference to perceptual or instructional implications.

### 2.4 Data extraction and analysis

**FIGURE 1:**
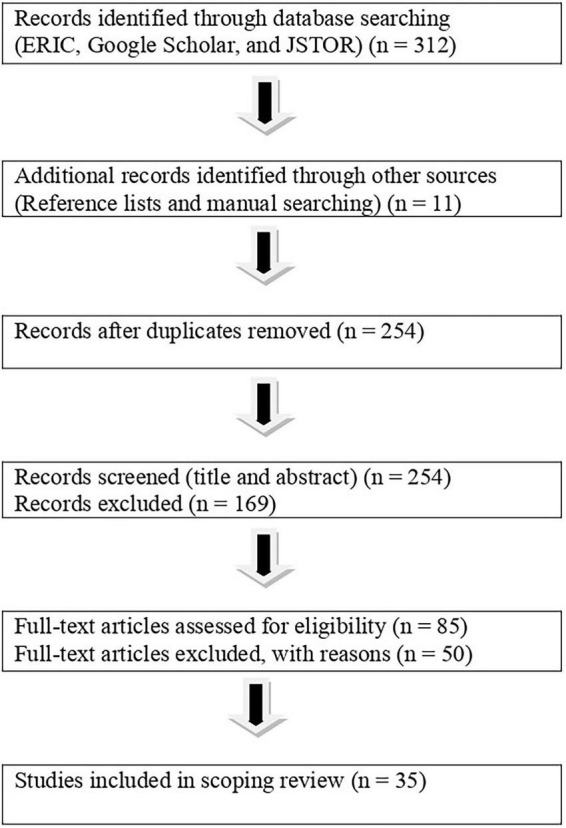
PRISMA-ScR flow diagram of the study selection process.

Comprehensive database searches yielded a substantial body of literature. After screening and eligibility assessment, 35 studies met the inclusion criteria and were retained for the final synthesis. The study selection process is illustrated using a PRISMA-ScR flow diagram (Figure 1).

Each source was coded and categorized by publication type, methodological orientation (experimental, descriptive, or theoretical), and principal conceptual contribution.

An iterative thematic synthesis was conducted to identify recurring constructs and organize them under three overarching domains:

1. *Perceptual-Cognitive Mechanisms*—studies examining auditory discrimination, tonal awareness, and interval processing;
2. *Pedagogical Modeling and Strategies*—works addressing instructional design, sequencing of exercises, and multisensory learning methods;
3. *Instructional Frameworks and Innovations*—contributions presenting novel models or visual-kinesthetic aids for solfege instruction (e.g., Curwen–Kodály method and Kendüzler’s method).

This tripartite framework provided the analytical foundation for interpreting how interval training connects perceptual cognition, pedagogical modeling, and practical instruction within solfege education.

## 3 The concept of interval

### 3.1 Definition and classification of intervals

According to Dowling and Harwood (1986), pitch is a fundamental perceptual element in both speech and music. In speech, pitch variations play a significant role in conveying meaning and emotion, whereas in music such variations form one of the primary components shaping melody. The magnitude of these changes is determined by the frequency ratio or the size of the interval between two sounds; in Western music, these intervals are expressed with distinctive names such as the minor third, perfect fifth, or octave (as cited in Zarate et al., 2012, p. 984).

Similarly, Crowder (1984), Narmour (1983), and Thompson (2009) emphasize that music is characterized by discrete transitions from one pitch to another—by intervals. The successive organization of these intervals constitutes melodic structure and carries structural, emotional, and aesthetic meanings (as cited in Thompson, 2013, p. 107).

In music theory, an interval is defined as the distance between two pitches. Intervals are categorized as harmonic or melodic depending on their written and performed forms: those sounded simultaneously or vertically notated are harmonic, while those performed successively or horizontally written are melodic. In addition, intervals within a single octave are classified as *simple*, whereas those exceeding one octave are considered *compound intervals* (Elhankızı, 2012, p. 61). Danhauser (1889) notes that the identification of intervals requires a systematic approach that evaluates both structural and auditory criteria.

The smallest distance between two adjacent pitches is defined as a *semitone*. Semitones are divided into chromatic and diatonic types: the chromatic semitone is produced by an alteration of the same pitch, while the diatonic semitone occurs between two notes with different letter names (Elhankızı, 2012, p. 62). In Western music, the octave is divided logarithmically into 12 equal parts. The distance between the notes C and D is called a *whole tone* (whole step), and the smallest perceptual unit within this system—half of a whole tone—is the semitone, representing one-twelfth of an octave (Levitin, 2006, pp. 29–30).

Intervals form the foundation of melodic structure and are defined not by the absolute pitch of the notes but by their relative distance. The ability to recognize different interval types facilitates students’ understanding of pitch relationships and melodic organization. Intervals in music range from narrow ones such as the minor second to wide ones such as the octave (see Table 1). Intervals are classified according to their number and quality: the number indicates how many scale degrees the interval spans, while the quality specifies the number of whole and semitone steps it contains. The numerical value written beside an interval denotes the number of scale degrees it encompasses (Elhankızı, 2012, p. 62).

**TABLE 1:** Simple musical intervals according to the number of semitones.

Number of semitones	Name of interval
0	Unison (same pitch)
1	Minor second
2	Major second
3	Minor third
4	Major third
5	Perfect fourth
6	Augmented fourth, diminished fifth, or tritone
7	Perfect fifth
8	Minor sixth
9	Major sixth
10	Minor seventh
11	Major seventh
12	Octave

Adapted from: this is your brain on music: the science of a human obsession by Levitin (2006).

### 3.2 Historical and theoretical perspectives

According to Levitin (2006, p. 29), the concept of an interval in music encompasses not only the frequency difference between two notes but also its perceptual interpretation. The octave, formed by doubling the frequency, stands out as a cross-cultural perceptual constant, suggesting that intervals are perceived through *relative relationships* rather than absolute frequency values. Backus (1969, p. 117) similarly explains that while an interval musically represents the distance between two notes—for instance, the spacing of two notes on a keyboard—it is physically defined as a ratio between frequencies. In this sense, interval perception is a multidimensional process that combines auditory recognition with cognitive processing of frequency ratios.

From a historical perspective, the study of musical intervals dates back to Pythagoras, who first demonstrated that consonant intervals arise from simple numerical ratios between string lengths—1:1, 2:1, 3:2, and 4:3—on the monochord. Because string length and frequency are inversely related, tones whose frequencies form such ratios are perceived as harmonious. Danhauser (1889, p. 42) later classified intervals according to their auditory stability and tendency toward resolution, distinguishing *perfect consonances* (octave, fifth), *imperfect consonances* (thirds and sixths), and *mixed consonances* (fourths, tritones) from *dissonances*, which demand resolution.

**FIGURE 2:**
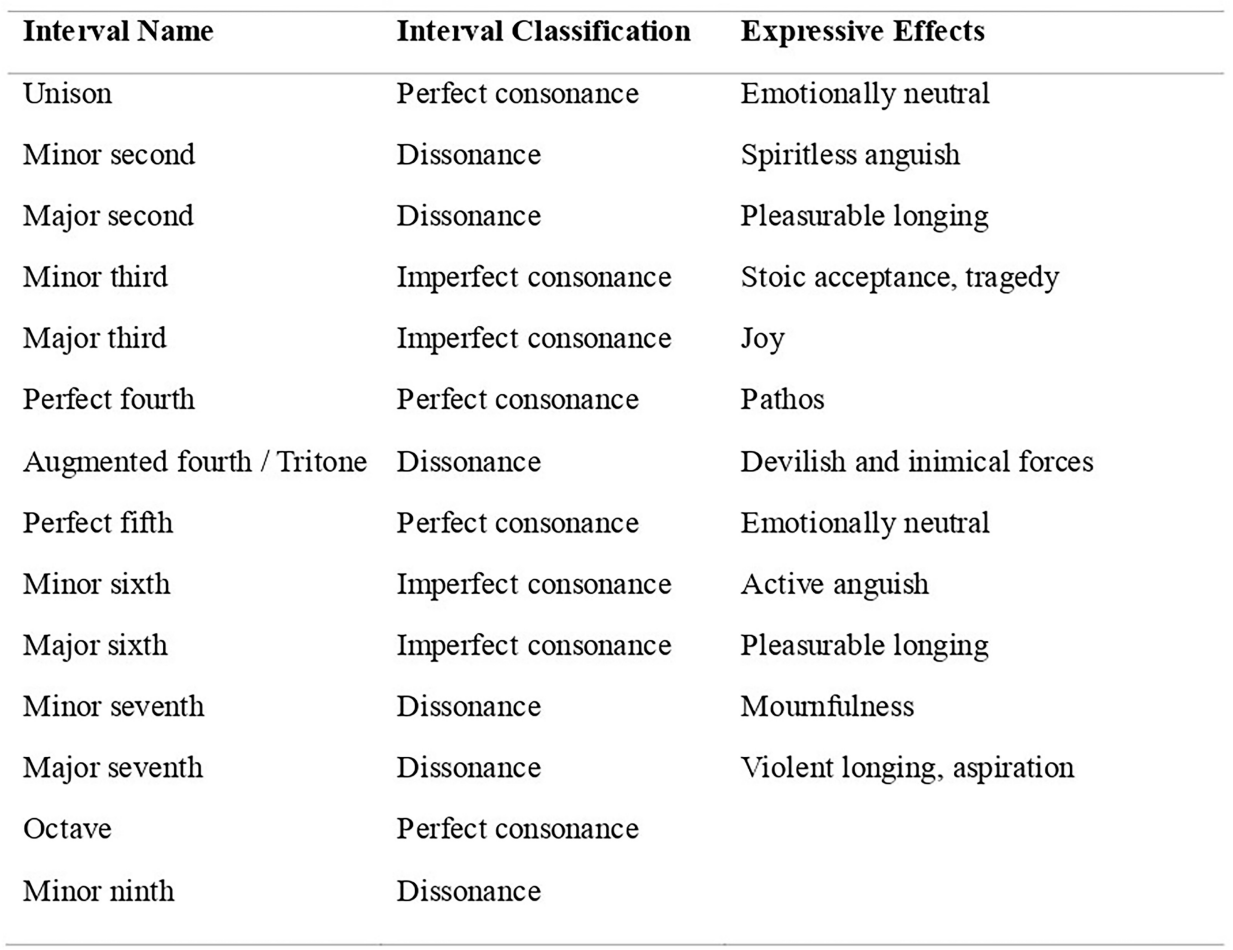
Interval classification and expressive effects of musical intervals. Adapted from Maher (1980).

Hevner (1935, p. 103) emphasized the affective role of intervallic structures in shaping musical meaning. She observed that professional musicians perceive distinct emotional differences between the major and minor modes: the major mode is typically associated with brightness and joy, while the minor mode conveys melancholy and introspection. Similarly, Maher (1980, p. 310) explored the expressive functions of musical intervals and confirmed that specific interval types evoke characteristic psychological effects, although these effects vary in strength across listeners (see Figure 2).

Maher (1980) reported that certain musical intervals produce distinct psychological effects and that his findings were generally consistent with prevailing views in the literature. However, nearly half of the intervals tested in his study could not be differentiated on any rating dimension, and only slightly more than half of the pairwise comparisons reached statistical significance. Therefore, although Maher’s research carefully demonstrated that musical intervals can influence psychological responses, it provided limited experimental evidence to support the widespread belief that each interval elicits a unique and consistent effect. In this sense, while the psychological impact of musical intervals cannot be entirely dismissed, it appears to be less clear and stable than traditionally assumed.

The evolution of interval perception also reflects shifts in theoretical interpretation. Schoenberg reframed consonance and dissonance not as opposites but as degrees of comprehensibility, arguing that dissonances are perceptually more complex rather than inherently unstable (as cited in Tenney, 1988, p. 2). Stravinsky further highlighted that dissonance possesses an autonomous aesthetic function, independent of its role in tonal resolution (as cited in Tenney, 1988, p. 3). Tenney concluded that the fluctuating definitions and perceptual reinterpretations of consonance and dissonance necessitate a historical-conceptual reconstruction of interval theory within Western music (as cited in Tenney, 1988, p. 3).

Together, these perspectives underscore that the concept of interval extends beyond acoustical measurement to encompass perceptual, cognitive, emotional, and historical dimensions. The interval thus represents not only a physical ratio between frequencies but also a multidimensional construct central to musical understanding and pedagogy.

Building upon the conceptual and historical foundations discussed above, the following sections present the thematic findings of this scoping review. The analysis identifies how perceptual-cognitive, pedagogical, and instructional dimensions intersect in the study of interval training within solfege education. While the concept of interval has long been theorized as both an acoustical and perceptual construct, recent empirical evidence expands this understanding by revealing its central role in auditory learning, melodic recognition, and intonation accuracy. Specifically, the findings address six interrelated domains: (1) perceptual-cognitive mechanisms underlying interval recognition, (2) the function of intervals as the building blocks of melodic structure, (3) memory and contour-related processes, (4) the effects of training and neural evidence, (5) tonal hierarchy and categorical perception, and (6) the sensory and cultural dimensions of consonance and dissonance. Together, these domains provide an integrated framework for understanding how interval perception supports the development of pitch cognition and musical learning in solfege pedagogy.

## 4 Findings and thematic synthesis

This section synthesizes the findings from 35 studies included in the review, organized across three thematic dimensions: (1) perceptual-cognitive mechanisms underlying interval recognition and pitch processing, (2) pedagogical modeling and instructional strategies in solfege-based training, and (3) innovative frameworks that integrate perceptual awareness with embodied and visual feedback. Together, these dimensions reveal how interval training functions as a multidimensional bridge between auditory perception, pedagogical design, and applied instruction in music education.

### 4.1 Perceptual-cognitive mechanisms underlying interval recognition

The perception of musical intervals arises from the interaction between auditory encoding, memory, and cognitive interpretation. Rather than processing isolated pitches, the auditory system organizes sounds into relational units that form the foundation of melody and tonality. Understanding how these mechanisms operate provides essential insight into how listeners recognize, categorize, and reproduce pitch distances across contexts.

This section outlines the principal perceptual and cognitive processes underlying interval recognition, including how intervals are represented as relative or absolute relations, how they function as the building blocks of melodic structure, how contour and magnitude are retained in memory, how training influences neural sensitivity, and how tonal hierarchy and consonance–dissonance shape perceptual organization. Together, these subtopics delineate the multidimensional nature of interval cognition and its relevance to solfege-based music learning.

#### 4.1.1 How the mind encodes musical intervals: relative and absolute representations of pitch

In perceptual terms, intervals are not experienced as static acoustic distances but as relational representations that link successive pitches. As Levitin (2006, pp. 29–30) explains, the ear encodes proportional frequency differences—ratios such as 2:1 (octave), 3:2 (perfect fifth), or 4:3 (perfect fourth)—which underlie the perception of tonal relationships. Within the 12-tone equal-tempered (12 EDO; Equal Divisions of the Octave) system, each semitone represents an equal logarithmic frequency ratio, producing the perceptual illusion of evenly spaced pitch steps.

This perceptual organization allows melodies to be recognized even when transposed: a major third interval (four semitones) retains its identity regardless of starting pitch, whether built on A, G#, or any other note (Levitin, 2006, p. 30). Consequently, musical perception is inherently relative in nature, prioritizing relationships between pitches rather than their absolute frequency values. The auditory system thus operates on a principle of relational constancy, preserving the pattern of pitch distances across tonal contexts.

By contrast, absolute pitch—the rare ability to label tones without external reference—relies on fixed pitch memory rather than relational mapping (Dowling and Harwood, 1986, as cited in Zarate et al., 2012, p. 984). While it represents a specialized form of auditory encoding, it does not constitute the primary mechanism for melodic perception. Instead, most listeners depend on relative encoding, which forms the cognitive foundation for melody recognition and tonal awareness (Crowder, 1984; Narmour, 1983; Thompson, 2009, as cited in Thompson, 2013, p. 107).

Pedagogically, this perceptual structure underlies solfege-based ear training, where students learn to perceive and reproduce intervals through relational pitch cognition. Emphasizing relative distance rather than fixed tone memorization fosters adaptability, tonal stability, and more accurate intonation in performance. This relational framework serves as the foundation for subsequent discussions on melodic structure, tonal hierarchy, and auditory learning mechanisms in solfege education.

#### 4.1.2 Intervals as building blocks of melodic structure

Intervals constitute the primary structural units through which melodies are organized and perceived. Crowder (1984), Narmour (1983), and Thompson (2009, as cited in Thompson, 2013, p. 107) emphasize that music is characterized by discrete pitch transitions—that is, by successive intervals that define melodic motion. The sequential organization of these intervals not only establishes melodic contour but also conveys emotional, structural, and aesthetic meaning. In this sense, melodic construction depends on the ordered succession of pitch relationships rather than on absolute tones, revealing the functional role of intervals as the essential “building blocks” of musical thought and expression.

According to Levitin (2006, p. 30), intervals form the foundation of melodic structure because the auditory system encodes relative distances between tones rather than their absolute frequencies. This relational encoding enables listeners to recognize melodies across transpositions: a major third retains its perceptual identity regardless of the starting note. Consequently, melodic perception operates through a system of relative pitch processing that preserves interval relationships even when pitch height changes. McDermott et al. (2010, p. 1943) similarly argue that music is perceived not by isolated tones but by the pitch changes between them, and that the brain represents these interval magnitudes independently of absolute pitch values. Such evidence underscores the cognitive primacy of intervallic organization in auditory pattern recognition.

Empirical findings also demonstrate that listeners encode both the contour and the exact interval sizes of familiar melodies. In a series of melodic memory experiments, Dowling and Fujitani (1971, pp. 530–531) found that participants could identify altered versions of known tunes more accurately when the relative magnitudes of successive intervals were preserved, even when transposed. This suggests that long-term melodic memory retains detailed intervallic information, not merely general directional shape. Thus, interval magnitudes serve as stable referents that anchor melodic identity and recognition.

Collectively, these findings confirm that intervals function as perceptual, cognitive, and expressive units that shape melodic understanding. They mediate the listener’s ability to organize tonal relationships, recall melodic sequences, and interpret musical meaning. Within solfege education, emphasizing interval awareness therefore cultivates the perceptual foundation upon which both melodic cognition and accurate vocal production depend, forming a bridge between auditory perception and performance accuracy.

#### 4.1.3 Memory, contour, and interval magnitude

Melodic memory research consistently shows that listeners encode not only the general contour of a melody but also the precise magnitudes of its constituent intervals. In one of the earliest experimental studies, Dowling and Fujitani (1971, pp. 530–531) tested whether melodies are remembered solely by their contour or also by the relative sizes of successive intervals. In short-term memory tasks, participants primarily relied on pitch information. However, when melodies were transposed, contour became a more dominant cue. In contrast, in long-term memory conditions, participants recognized altered versions of familiar folk tunes more accurately when both contour and relative interval sizes were preserved. These findings suggest that long-term melodic representations retain not only directional shape but also fine-grained intervallic detail.

Similarly, White (1960, p. 103) demonstrated that altering interval magnitudes within a melody significantly impairs recognition, even when overall contour remains intact. Transformations preserving both the contour and interval sizes were recognized most reliably, while those maintaining contour but modifying interval magnitudes disrupted melodic identification. These results indicate that interval magnitude serves as a stable perceptual anchor in melodic recognition, complementing the broader contour information.

From a cognitive standpoint, McDermott et al. (2010, p. 1943) argue that music is perceived not by absolute tones but by the pitch changes between them. The auditory system represents these changes as abstract interval relationships that remain invariant under transposition. This invariance explains why a familiar melody can be recognized regardless of the starting pitch—the relational encoding of intervals allows the listener to reconstruct melodic identity based on interval sequences rather than absolute frequencies.

Collectively, these findings highlight the complementary roles of contour and interval magnitude in melodic perception. While contour provides a global schematic of pitch direction, interval magnitude ensures precision and stability in memory. Within solfege education, this dual encoding underscores the pedagogical necessity of training both relational awareness and fine interval discrimination. Structured interval practice thus strengthens the integration of auditory memory, contour processing, and melodic reproduction—key components in developing accurate intonation and musical fluency.

#### 4.1.4 Training effects and neural correlates of interval perception

Empirical research provides compelling evidence that interval perception is shaped and refined through musical training. Studies in auditory neuroscience and psychophysics have shown that musicians exhibit greater sensitivity to subtle pitch differences and demonstrate superior accuracy in identifying intervals across tonal contexts. Specifically, Zarate et al. (2012, pp. 991–992) reported that musical training enhances discrimination thresholds for pitch intervals, with musicians reliably identifying differences as small as 100 cents, whereas non-musicians require larger pitch distances to achieve comparable accuracy.

Neuroimaging data further reveal that increasing interval size is associated with heightened activation in the auditory cortex and the intraparietal sulcus (IPS)—regions responsible for integrating sensory and cognitive computations during auditory processing. These findings indicate that interval perception involves a distributed neural network that coordinates bottom-up acoustic encoding with top-down cognitive evaluation. The activation of such distributed areas suggests that musical expertise not only refines auditory acuity but also recruits additional cortical mechanisms for monitoring pitch distance and tonal relationships.

From a pedagogical standpoint, this neural adaptability underscores the role of systematic interval training in strengthening both perceptual and cognitive dimensions of musicianship. Structured solfege exercises—particularly those emphasizing fine interval discrimination and consistent auditory feedback—can stimulate similar neural plasticity, enabling learners to internalize stable pitch representations and achieve greater intonational precision.

In essence, interval perception is not a fixed sensory capacity but a trainable skill grounded in experience-dependent neural reorganization. The evidence that the brain dynamically adjusts to the statistical regularities of musical input supports the pedagogical rationale for sustained solfege training. Through repeated practice, learners refine their perceptual thresholds, enhance auditory-motor synchronization, and build the cognitive infrastructure necessary for accurate melodic performance.

#### 4.1.5 Tonal context, hierarchical organization, and categorical perception of intervals

Tonal structure provides a cognitive framework that shapes how listeners encode, predict, and reproduce pitch intervals. Graves and Oxenham (2017, p. 1) found that individuals exhibit stronger interval recognition accuracy when tones are presented within familiar tonal frameworks (e.g., diatonic or major scales) compared to atonal contexts. This effect suggests that tonal settings activate stable pitch representations in auditory memory, allowing listeners to compare interval distances more efficiently. Rather than resulting from improved sensory resolution, this facilitation likely stems from tonal expectations that prime the auditory system for predicted pitches and harmonic functions. Tonal structure is based on specific tendencies that guide melodic and harmonic motion and generate expectations in the listener (Siegmeister, 1965; Ratner, 1966; Piston, 1978; Schenker, 1935, as cited in Schmuckler, 1989, p. 111).

Supporting this view, Holmes (2009, p. 55) noted that experienced sight-readers do not perceive notes as isolated entities but as organized tonal patterns. During reading or singing, such internalized tonal hierarchies guide the perception of interval direction and size, promoting accuracy in intonation and melodic reproduction. Similarly, Grutzmacher’s (1985, as cited in Holmes, 2009, pp. 57–58) findings showed that tonal pattern instruction—through harmonization and vocalization—significantly improved beginner students’ ability to sight-read and discriminate between major and minor tonalities, highlighting the pedagogical value of embedding interval exercises within tonal contexts.

From a cognitive standpoint, tonal structures operate through statistical learning mechanisms. Listeners unconsciously internalize recurring tonal relationships and scale patterns through exposure, which in turn facilitate perceptual fluency. Graves and Oxenham (2017, p. 2) interpreted this as a form of perceptual priming, whereby salient tones within a tonal hierarchy become stronger reference anchors in auditory memory. Such reinforcement allows learners to identify intervals not as isolated acoustic distances but as functional pitch categories relative to a tonal center.

In solfege instruction, this phenomenon corresponds to categorical perception, in which discrete pitch relationships (e.g., “major third,” “minor third”) are recognized as perceptually stable categories rather than as continuous acoustic values. As Özaltunoğlu (2003, p. 2) explained, solfege students mentally visualize each tone’s function within the scale, constructing an internal “graphic” of tonal relationships before vocalizing. This process reflects how tonal context transforms abstract pitch distances into meaningful musical categories.

Pedagogical evidence also supports combining tonal-centered and scale-based instruction. Jarjisian (1981, as cited in Holmes, 2009, p. 57) found that students trained with both pentatonic and diatonic materials demonstrated higher song memorization accuracy and tonal awareness than those exposed to a single system. Diatonic training reinforced the sense of tonal center, while pentatonic exercises enhanced contour sensitivity—together fostering a more flexible and comprehensive understanding of melodic structure.

Collectively, these findings emphasize that tonal hierarchy functions as a perceptual scaffold for interval learning. Embedding interval exercises within tonal and functional frameworks strengthens categorical pitch perception, stabilizes intonation, and enhances melodic predictability. For solfege pedagogy, this underscores the importance of cultivating both absolute tonal orientation and relative interval recognition—skills that interact to produce accurate, context-sensitive musical performance.

#### 4.1.6 Consonance and dissonance: sensory and cultural factors

Perceptually, consonance and dissonance represent two fundamental dimensions through which the auditory system organizes interval relationships. Backus (1969, p. 116) defined consonance as the combination of two or more tones whose frequencies produce a pleasing and balanced auditory effect, whereas dissonance evokes tension and the expectation of resolution. Similarly, Danhauser (1889, p. 42) classified intervals according to their perceptual stability: perfect consonances (octave, perfect fifth), imperfect consonances (thirds and sixths), and unstable dissonances that demand resolution. These classifications indicate that the sense of consonance arises from both acoustic regularity and perceptual equilibrium.

Empirical evidence demonstrates that the preference for consonant combinations may be rooted partly in innate sensory mechanisms. Studies show that even infants and some animal species exhibit spontaneous preferences for consonant tone pairs over dissonant ones Trainor et al., 2002; Hannon and Trainor, 2007; Chiandetti and Vallortigara, 2011; McDermott and Hauser, 2005 (as cited in Thompson, 2013). These findings suggest that the aesthetic appeal of consonance is not merely culturally learned but grounded in neurobiological predispositions. Nevertheless, such preferences are also shaped through exposure and enculturation, as prolonged interaction with particular tonal systems refines what listeners perceive as pleasant or tense.

From a cognitive perspective, consonance and dissonance operate as affective cues that convey musical motion and emotional valence. Maher (1980, p. 310) demonstrated that specific intervals elicit distinct psychological responses, aligning broadly with theoretical expectations; however, many comparisons failed to reach statistical significance, implying that affective responses to intervals are not universal but context-dependent. Consequently, while consonance often evokes stability and brightness and dissonance signals tension or introspection, their perceptual effects vary across cultural frameworks and compositional idioms.

Historically, the conceptual boundary between consonance and dissonance has shifted with musical practice. As Tenney (1988, pp. 2–3) observed, intervals once considered dissonant—such as the third—later acquired consonant status as tonal systems evolved. Theories by Hindemith and Schoenberg reframed these categories not as opposites but as degrees of perceptual comprehensibility, arguing that dissonance embodies greater complexity rather than inherent unpleasantness. This reinterpretation paved the way for modernist aesthetics in which dissonance serves expressive, not merely functional, purposes. Such historical flexibility underscores that the perception of consonance and dissonance cannot be understood solely through acoustics but must be contextualized within cultural and stylistic evolution.

In sum, the interplay between sensory universals and cultural particularities defines how intervals are evaluated and experienced. While the biological preference for simple frequency ratios provides a perceptual anchor, cultural exposure determines which intervallic structures are perceived as consonant or dissonant. For solfege education, acknowledging this dual nature is pedagogically significant: teaching consonance and dissonance as perceptual continua—rather than fixed binaries—can help students appreciate both tonal stability and expressive tension as integral components of musical meaning.

### 4.2 Pedagogical modeling and strategies

Pedagogical modeling in solfege education integrates perceptual awareness, motor learning, and instructional design to develop stable pitch perception and accurate vocal production. Across the reviewed studies, six interrelated pedagogical strategies emerged: (1) interval-centered ear–voice coordination; (2) practice sequencing for skill retention; (3) use of external auditory references; (4) associative learning through reference melodies; (5) tonal-centered instruction grounded in functional pitch relationships; and (6) technique-based approaches that combine systematic drills with exposure-based learning. Collectively, these pedagogical models demonstrate how structured auditory-motor interaction supports the integration of perceptual and behavioral mechanisms that underlie pitch accuracy, tonal awareness, and the acquisition of musical expertise.

#### 4.2.1 Functional role of interval training in aural and vocal skills

Interval training functions as a cornerstone of aural-vocal education by reinforcing the link between pitch perception and vocal production. Lake (1993, pp. 55–58) identified two complementary perceptual strategies: interval-oriented listening, where learners focus on the acoustic distance between tones, and scale-degree-oriented listening, in which tones are related to a tonal center. Early stage learners typically rely on the first, while advanced musicians integrate both for flexible tonal processing.

Killian and Henry (2005, pp. 55–64) demonstrated that students with consistent interval practice show superior intonation accuracy, improved rhythmic stability, and better self-correction habits. These learners employ metacognitive rehearsal techniques such as singing isolated phrases, marking problem notes, and using hand signs to coordinate sound and gesture. Such findings highlight interval training as a perceptual and behavioral bridge between auditory recognition and vocal-motor execution, supporting accurate tuning and expressive control in solfege education. This dual emphasis on perceptual awareness and motor control exemplifies the integrative nature of auditory learning as conceptualized in contemporary music cognition research.

#### 4.2.2 Practice design: blocked and interleaved learning

The organization of practice—whether blocked or interleaved—has a marked influence on perceptual retention and transfer. Wong et al. (2021, p. 1027) found that blocked practice (repetition of a single interval type) enhances immediate accuracy for beginners, while interleaved practice (alternating between multiple interval types) improves long-term retention and adaptability. Learners exposed to interleaved sequences exhibited better discrimination of novel melodic patterns and greater resistance to interference.

Pedagogically, combining both formats produces optimal results: blocked practice consolidates early accuracy, whereas interleaved exposure fosters flexibility and transfer across tonal contexts. This hybrid sequencing promotes sustainable auditory generalization—an essential goal of solfege-based interval training. This adaptive interplay between blocked and interleaved sequencing reflects the principles of contextual interference, suggesting that variability in practice enhances neural plasticity and perceptual robustness in tonal learning.

#### 4.2.3 External references and accompaniment: piano as an aural anchor

External references, particularly piano accompaniment, serve as aural anchors that support learners’ pitch calibration. As Atterbury and Silcox (1993, p. 41) and Mariner and Schubert (2021, p. 129) note, the piano’s fixed tuning and visual spacing provide an immediate framework for conceptualizing intervallic distances. The instrument also functions as a multimodal scaffold—linking auditory, visual, and kinesthetic domains—that strengthens learners’ pitch–interval mapping.

However, pedagogues such as Curwen and Kodály warned that excessive dependence on external references may inhibit the development of internal audiation. Therefore, the piano should act as a transitional aid, gradually withdrawn as learners acquire self-regulated pitch awareness and vocal independence. From a perceptual–cognitive perspective, such gradual withdrawal of external anchors promotes the neural transition from stimulus-dependent tuning to internally generated pitch representation.

#### 4.2.4 Reference melodies and associative scaffolds

**FIGURE 3:**
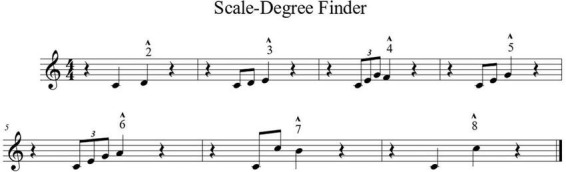
Scale-degree finder. Adapted from Lake (1993).

Associating familiar melodies with specific intervals enhances recognition and recall during training. Lake (1993, p. 60) emphasized that short melodic phrases containing target intervals provide auditory anchors that facilitate rapid identification. These exercises allow learners to verify their guesses by recalling or vocalizing the relevant passage whenever they encounter difficulty (see Figure 3).

Similarly, Smith et al. (1994) observed that students using familiar tunes as reference performed significantly better in interval detection tasks (as cited in Wong et al., 2021, p. 1030).

**FIGURE 4:**
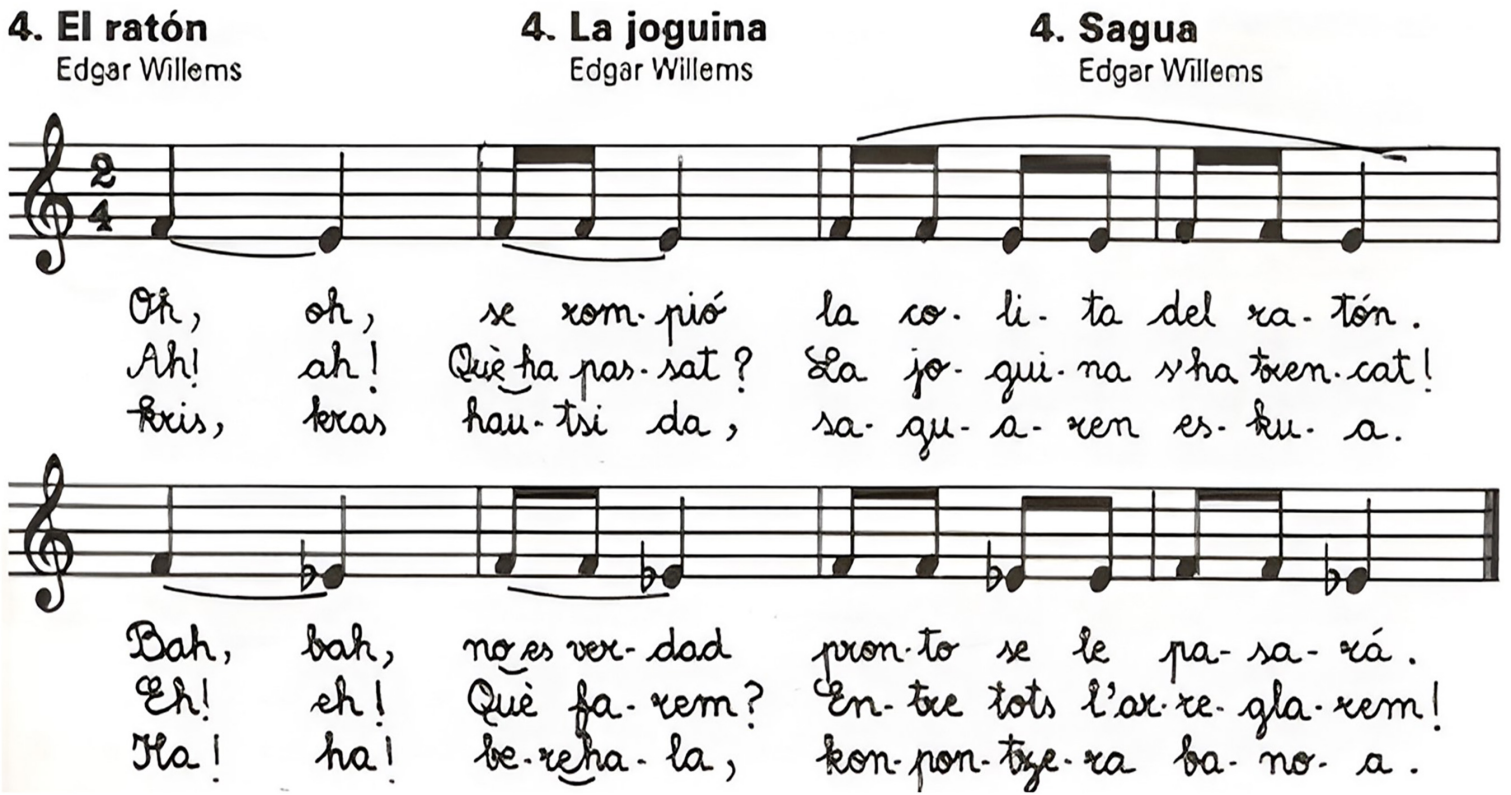
Examples of reference songs used to identify interval qualities (El ratón, La joguina, Sagua; from Edgar Willems). Source: Chapuis and Willems (1996).

In this context, linking a known song’s opening phrase with a specific interval can meaningfully support students in recalling both the size and the quality of intervals, while strengthening their aural identification skills. An example of such an approach, showing the descending forms of a familiar tune used to teach seconds, is presented below (see Figure 4).

Further applications of the AIMHI model (Ponsatí et al., 2016) demonstrate that singing familiar songs while visualizing interval distances promotes internal hearing and strengthens memory consolidation. Yet, as Karpinski, 2000; Rogers, 2004 (as cited in Wong et al., 2021, s. 1031). caution, educators should gradually reduce reliance on mnemonic songs to foster autonomous interval perception and self-generated tonal imagery. This gradual transition from external mnemonics to internal tonal imagery reflects the perceptual–cognitive shift central to adaptive musical learning.

#### 4.2.5 Tonal-centered training (movable-do approach)

A tonal-centered framework—especially the movable-do system—places intervals within functional pitch contexts, emphasizing their roles in harmonic and melodic progression. Erol (2019) and Bentley (1959) highlight that interval comprehension improves when learners understand the tonal hierarchy governing pitch relationships. By consistently relating intervals to the tonic, students internalize both direction and harmonic function.

Koga (2016) and Voth (2006) further found that tonal-centered solfege exercises enhance cognitive encoding of pitch stability and promote flexible transposition skills. This orientation integrates theoretical understanding with auditory experience, aligning solfege instruction with tonal cognition rather than abstract distance memorization.

**FIGURE 5:**
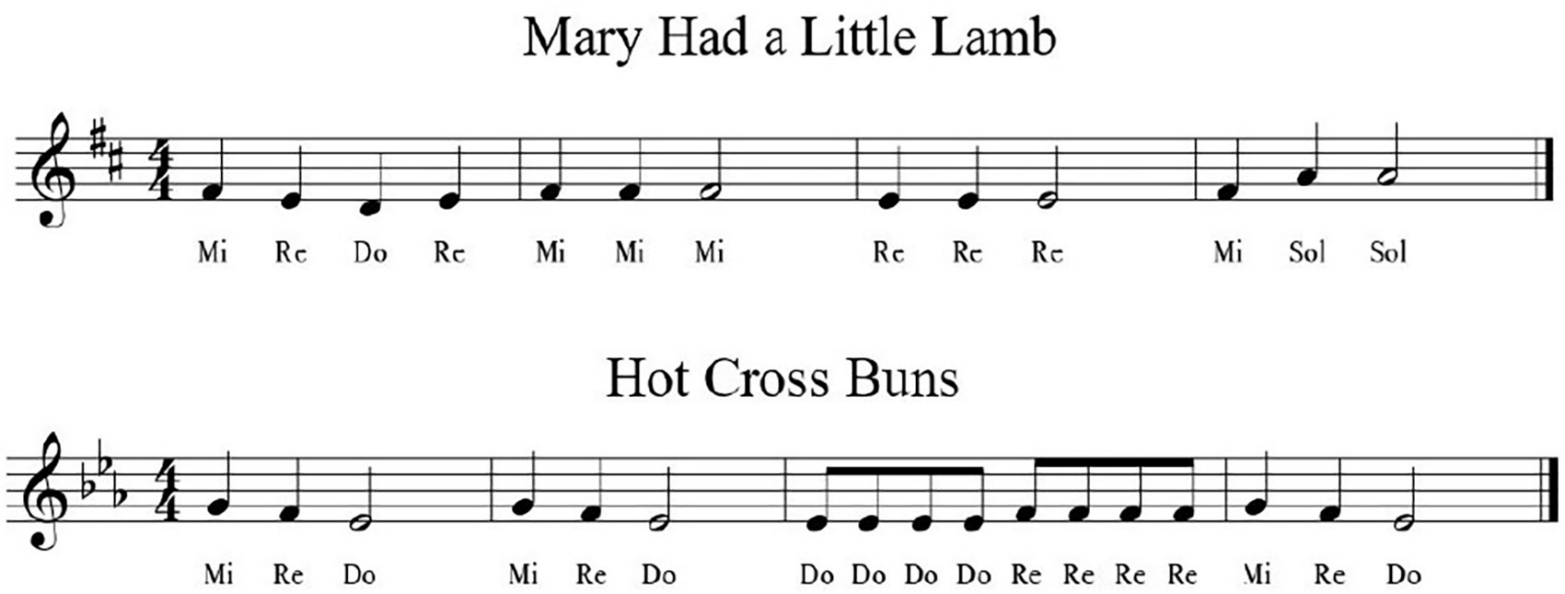
Two children’s songs written using the movable-do system. Adapted from Koga (2016).

To illustrate the application of the movable-do system, two examples of children’s songs are presented below (see Figure 5). From a perceptual-cognitive perspective, this tonal orientation stabilizes pitch memory through hierarchical encoding, enabling flexible generalization across keys.

#### 4.2.6 Technique-based drills and exposure-enhanced learning

Finally, contrasting methodologies in interval pedagogy reveal two complementary paradigms: technique-based drills, focusing on systematic repetition, and exposure-enhanced learning, emphasizing varied auditory input. Little et al. (2019, p. 344) found that combining structured practice with passive listening improved pitch discrimination, interval identification, and transfer to novel melodies. Students who engaged in both active and exposure-based learning demonstrated more consistent intonation and shorter adaptation times when performing.

This convergence of structured technique and immersive exposure forms the basis for sustainable auditory skill development. Pedagogically, alternating between deliberate, focused drills and varied listening environments fosters both precision and flexibility—key components of expert-level solfege competence.

Collectively, the six pedagogical models demonstrate that interval training in solfege education is not a singular exercise method but a multidimensional pedagogical system. Effective instruction balances structured practice with adaptive exposure, and external scaffolds with internal audiation. These models reveal how auditory, visual, and kinesthetic modalities interact to stabilize pitch accuracy and tonal cognition. The integration of such pedagogical strategies provides a foundation for innovative instructional frameworks that link perception, physiology, and visualization—discussed in the following section. Such integrative designs mirror the neural coupling of auditory and motor systems that underpin adaptive musical expertise.

### 4.3 Instructional frameworks and innovations

#### 4.3.1 Hand signs as spatial-visual mapping

**FIGURE 6:**
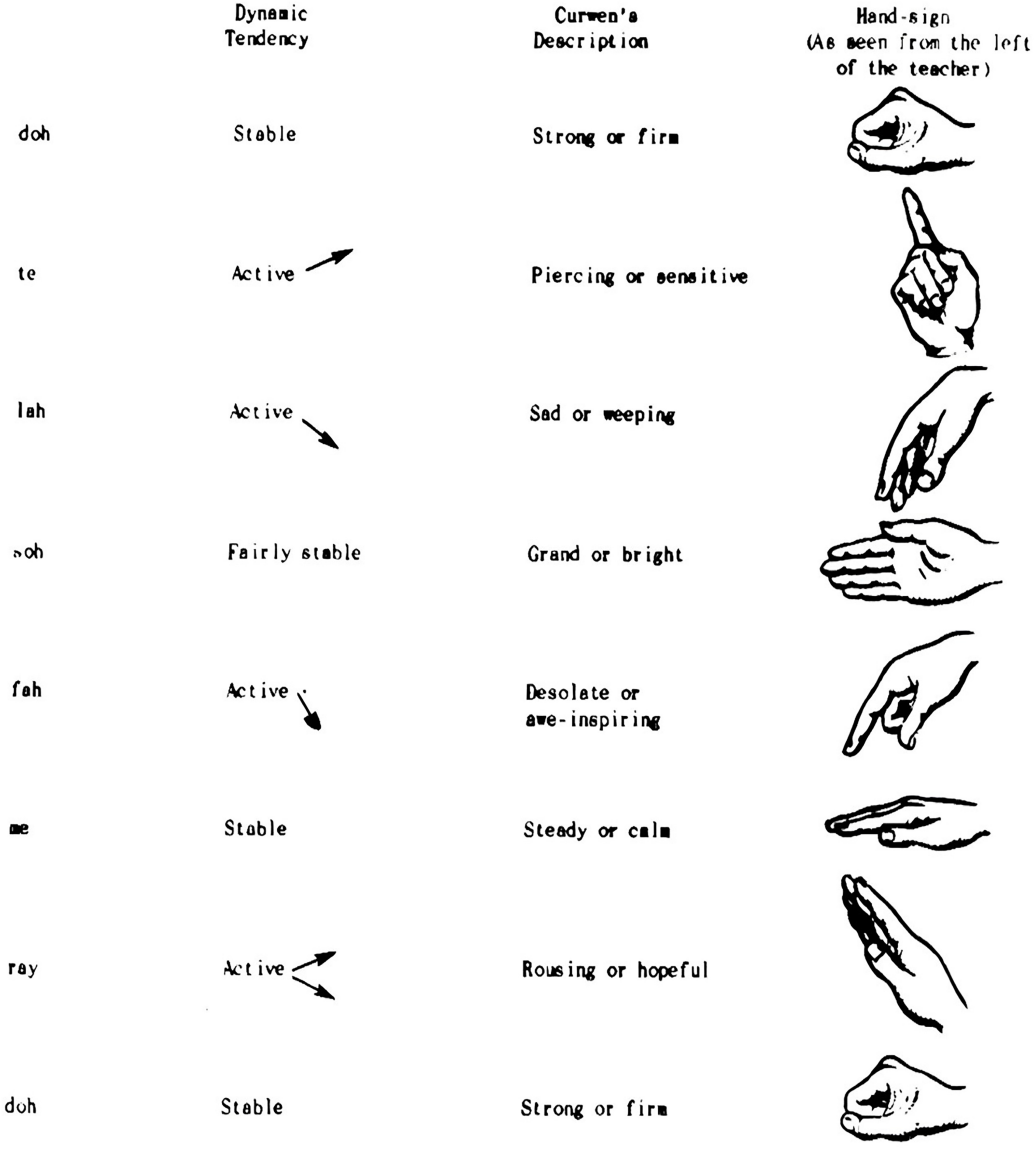
Curwen hand signs and their associated mental effects. Source: Steeves (1984).

**FIGURE 7:**
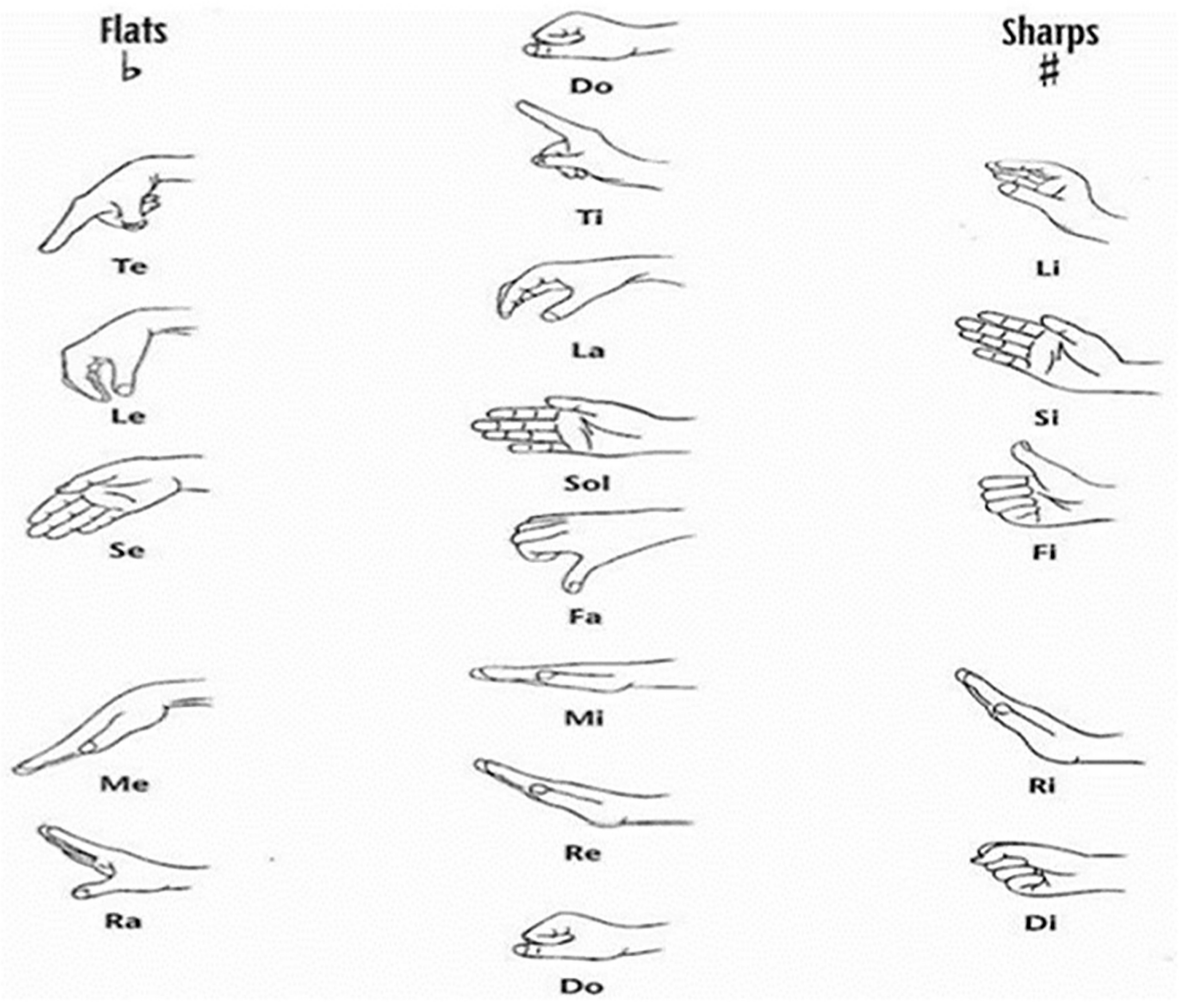
Hand signs adapted by Zoltán Kodály. Source: Earl Haig Secondary School (n.d.).

Hand signs have historically played a vital role in music pedagogy, serving as a visual-kinesthetic bridge between notation, sound, and physical gesture. Among the earliest examples is cheironomy—the ancient practice of guiding melodic contour through hand movements—which emerged in Egypt and Byzantium and was later adapted across various cultural traditions (Gerson-Kiwi, 1980, as cited in Steeves, 1984, p. 2). The modern system of hand signs owes its development to John Curwen (1816–1880), who designed a set of gestures to visually represent pitch height and expressive character. Curwen’s system was later refined and expanded by Zoltán Kodály, who integrated it into his comprehensive method for teaching tonic solfa through multisensory engagement (Steeves, 1984, pp. 1–2; see Figures 6, 7).

The Curwen-Kodály system assigns a distinct hand sign to each scale degree, positioned vertically along the body to symbolize pitch direction. Instruction typically begins with *do* at waist level and ascends stepwise until *ti* reaches eye level (Earl Haig Secondary School, n.d.). This mapping transforms abstract pitch relationships into spatially and kinesthetically accessible representations, reinforcing internal audiation through movement. Each gesture embodies not only the pitch’s position but also its expressive “personality,” as Curwen believed that tones exert unique emotional and dynamic tendencies (Simpson, 1973, as cited in Steeves, 1984, p. 9).

Empirical and pedagogical evidence suggests that such multimodal reinforcement strengthens students’ tonal memory, pitch stability, and interval recognition. By coupling motor and auditory feedback, learners can visualize harmonic shifts, modulations, and chromatic alterations through changes in hand position or orientation (Curwen, 1875, as cited in Steeves, 1984, pp. 11–12). Moreover, Curwen’s colleague Cullingford demonstrated that both hands could be used simultaneously to indicate polyphonic or modulatory movement, allowing complex tonal relations to be visually internalized.

In solfege education, hand signs therefore serve as an embodied form of cognitive scaffolding—transforming pitch relationships into spatial-visual schemas that support perception, memory, and expressive interpretation. The Curwen-Kodály model exemplifies how visual and kinesthetic modalities can complement auditory training, establishing a perceptual foundation for more integrated and physiologically guided instructional frameworks. This embodied mapping supports sensorimotor integration in pitch processing, providing a dynamic interface between perception and action in tonal learning.

#### 4.3.2 Physiological feedback framework: diaphragm-guided crescendo-decrescendo

**FIGURE 8:**
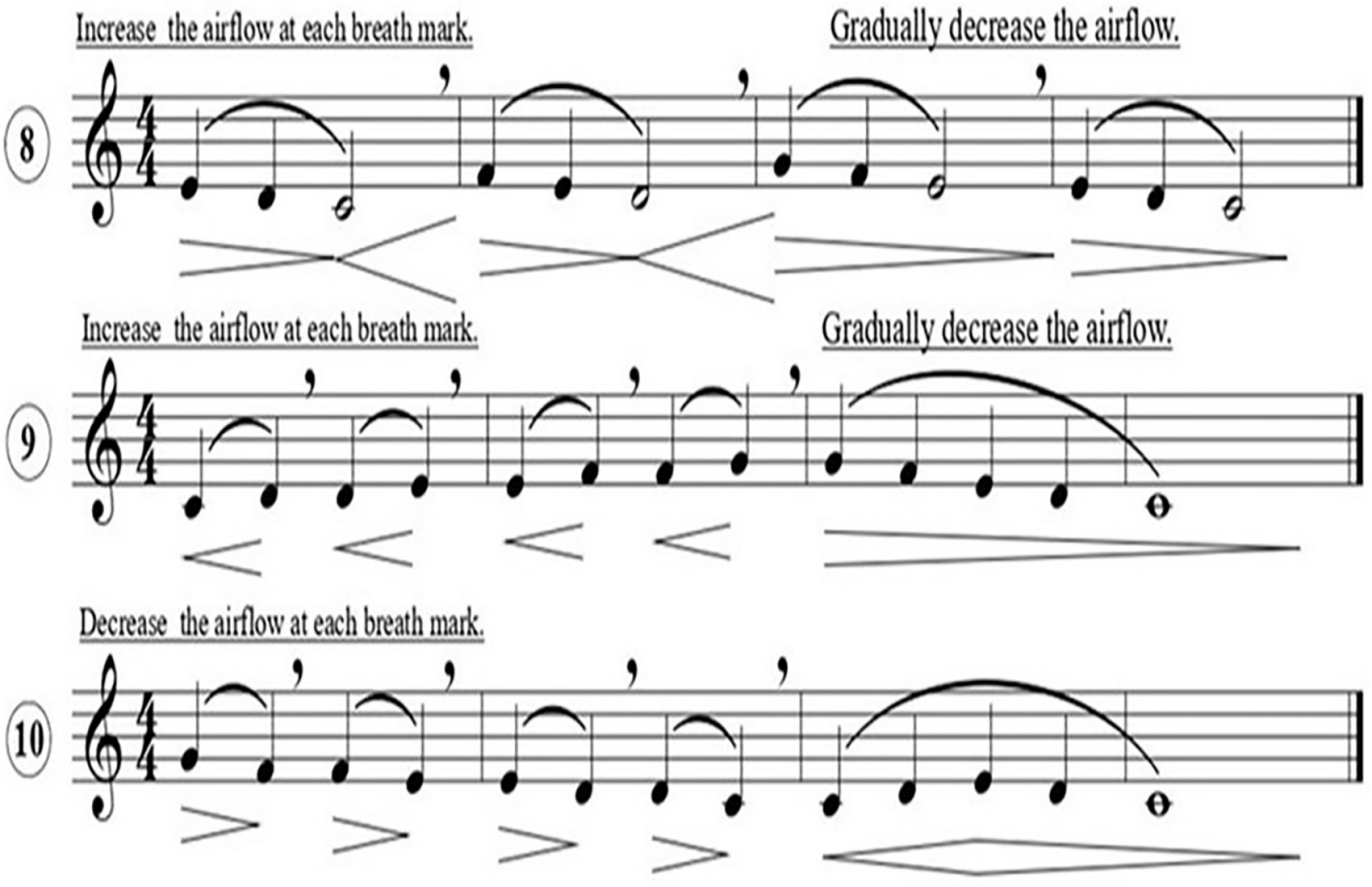
Crescendo and decrescendo markings indicating diaphragmatic tension according to interval distance. Adapted from Kendüzler and Akkas (2025).

The *Vocal Use-Based Solfege Teaching Model* (Kendüzler, 2023) represents a recent pedagogical innovation that integrates physiological feedback into the teaching of musical intervals. Grounded in principles of diaphragmatic control, this method visualizes intervallic distance through varying slopes of crescendo and decrescendo indicators positioned beneath each exercise (see Figure 8).

Unlike conventional dynamic markings, these indicators symbolize the degree of diaphragmatic tension required for accurate pitch production rather than changes in volume. When the interval between two pitches is wider, the crescendo line is drawn with a steeper slope to signify greater diaphragmatic engagement; when the interval is narrower, the slope becomes more horizontal, reflecting reduced tension demands. This visual-kinesthetic mapping transforms the invisible process of breath regulation into a perceivable guide for vocal control. As students sing, the slope functions as a real-time feedback cue, allowing them to align respiratory effort with intervallic spacing and melodic direction.

Pedagogically, the model advances the concept of *embodied audiation*—the integration of sensory perception, vocal production, and physiological regulation. By linking visual cues to muscular activity, learners internalize the mechanics of pitch generation, thereby improving intonation accuracy and breath coordination. Furthermore, the system’s progressive design accommodates both individual and group learning contexts: its scalable structure allows exercises to be simplified or expanded according to vocal range and developmental level without compromising pedagogical integrity.

From a theoretical perspective, this approach bridges perceptual and motor domains, aligning with embodied cognition theories that emphasize the role of sensorimotor processes in abstract learning. It extends traditional fixed-do solfege pedagogy by introducing a physiological dimension, converting internal tension into a visualized parameter. The result is a holistic instructional framework in which perception, movement, and expression coalesce into a unified system of learning and performance. This alignment between physiological feedback and perceptual awareness mirrors findings in auditory-motor research, where respiratory control is shown to modulate pitch precision.

Taken together, these instructional frameworks reveal a progressive continuum in solfege pedagogy—from externalized visual aids to embodied physiological feedback. The Curwen–Kodály system translates pitch relationships into spatial and kinesthetic gestures, while the diaphragm-guided model transforms vocal mechanics into visualized feedback loops. Both approaches demonstrate that effective solfege instruction relies on the integration of perception, movement, and controlled expression rather than on isolated listening skills. This synthesis reflects an evolution from symbolic to embodied learning, showing how interval cognition can be strengthened when sensory, cognitive, and motor dimensions operate in concert. These interconnections lay the conceptual foundation for the ensuing discussion, where perceptual and pedagogical insights are examined in light of contemporary theories of musical learning and performance.

## 5 Discussion

This scoping review set out to clarify how interval training supports pitch cognition and musicianship in solfege education by synthesizing evidence across three axes: (1) perceptual-cognitive mechanisms, (2) pedagogical modeling, and (3) instructional innovation. Taken together, the literature indicates that intervals function as relational units encoded by the auditory system, and that targeted training strengthens the mapping between perception and vocal production. Pedagogically, effective programs balance structured practice with adaptive exposure and embed intervals within tonal frameworks, while innovative visual-kinesthetic tools can externalize otherwise covert auditory-physiological processes. These perspectives collectively situate solfege as a model system for studying the interaction between perception, action, and cognition in musical learning. Below, we discuss implications and open questions along these axes.

### 5.1 Perceptual-cognitive implications

The convergent evidence that listeners encode intervallic relations (rather than absolute pitch values) explains why melodies remain recognizable under transposition and why fine interval magnitudes, not only contour, stabilize melodic identity. Importantly, learners appear to benefit from dual strategies: (a) interval-oriented listening that tracks specific distances and (b) scale-degree-oriented listening that references a tonic center. Flexibly coordinating these strategies yields greater resilience across contexts (e.g., tonal vs. atonal, familiar vs. unfamiliar materials). Moreover, findings on tonal hierarchy suggest that exposure-driven expectations prime categorical pitch judgments; teaching consonance-dissonance as a continuum rather than a fixed binary can help students integrate both stability and expressive tension into performance. This aligns with predictive coding accounts of auditory cognition, where tonal expectations shape both perceptual stability and expressive nuance.

### 5.2 Pedagogical implications

Instructionally, interval training acts as a bridge between auditory recognition and vocal–motor execution. Three design principles recur.

**Practice sequencing:** A hybrid of blocked (early accuracy) and interleaved (retention and transfer) practice optimizes learning.

**Scaffolds with fading:** External anchors (piano, reference melodies, hand signs) accelerate calibration and memory, but should be systematically withdrawn to cultivate internal audiation.

**Contextualization:** Placing intervals in tonal frameworks (e.g., movable-do) reinforces functional pitch relations and supports transposition skills.

Collectively, these choices promote accurate intonation, error detection and recovery, and durable generalization to new melodic contexts. Such sequencing and scaffold management exemplify adaptive instruction rooted in perceptual feedback, aligning teaching design with cognitive models of musical skill acquisition.

### 5.3 Instructional innovations and embodied learning

Visual-kinesthetic approaches translate abstract pitch relations into perceivable forms. The Curwen-Kodály hand-sign system maps scale degrees onto space and gesture, strengthening categorical perception and supporting modulation tracking in real time. Complementarily, the Vocal Use-Based Solfege Teaching Model, developed by the author, introduces a physiological feedback channel: crescendo/decrescendo slopes encode diaphragmatic tension requirements proportional to interval span. Whereas Curwen-Kodály emphasizes the expressive “personality” and spatial logic of degrees, the author’s model foregrounds breath mechanics and motor control. In combination, these frameworks exemplify how embodied design can tighten the perception-action loop that underwrites intonation accuracy. This embodied continuum—from spatial-visual mapping to physiological feedback—illustrates how multimodal coupling can foster both expressive awareness and motor precision in tonal performance.

### 5.4 Limitations of the evidence base

Despite consistent trends, the literature shows heterogeneity in (a) outcome measures (discrimination thresholds, sight-singing accuracy, categorical judgments), (b) training dosages and durations, and (c) participant profiles (musical background, age). Many studies rely on short interventions and small samples; Western-centric materials predominate, limiting cultural generalizability. Experimental contrasts between practice schedules or scaffolds are often underpowered, and few studies track long-term maintenance. Addressing these limitations will require longitudinal, cross-cultural, and ecologically valid designs that connect laboratory findings with authentic educational settings.

### 5.5 Directions for future research

Future work should deploy longitudinal, adequately powered trials comparing blocked and interleaved sequencing as well as scaffold-fading protocols; test transfer to authentic performance contexts such as ensemble tuning and sight-singing under time pressure; examine cross-cultural materials (e.g., pentatonic, pelog) to probe perceptual universals vs. enculturation; integrate neurophysiological indices such as cortical tracking of interval size and audio-motor coupling during training; and evaluate embodied frameworks—including hand signs and diaphragm-guided cues—through formal usability and learning-curve analyses.

In sum, interval-based training provides a multidimensional substrate for solfege education: it refines perceptual resolution, stabilizes tonal orientation, and concretizes the sensorimotor control required for accurate vocal production. Programs that integrate relational listening, tonal functionality, and embodied scaffolds are best positioned to cultivate durable, transferable musicianship. Bridging these lines of inquiry will clarify how perceptual, cultural, and physiological factors jointly shape the acquisition of musical pitch expertise.

## 6 Conclusion

This scoping review synthesized evidence on how interval-based training supports pitch cognition and musicianship in solfege education across three complementary levels: perceptual–cognitive mechanisms, pedagogical modeling, and instructional innovation. The literature consistently indicates that the auditory system encodes relational interval structures rather than absolute pitch values, that contour and precise interval magnitudes jointly stabilize melodic identity, and that tonal hierarchies scaffold categorical judgments and intonation. These mechanisms explain why structured interval practice—particularly when embedded in tonal contexts—improves pitch discrimination, error detection, and vocal-motor coordination.

Pedagogically, the most effective programs balance blocked and interleaved practice, pair external scaffolds (piano anchors, reference melodies, hand signs) with planned scaffold fading to cultivate internal audiation, and align exercises with functional (movable-do) frameworks to reinforce transposition and tonal orientation. Innovative visual-kinesthetic approaches further enhance learning by externalizing covert processes: Curwen-Kodály hand signs spatialize scale-degree “personality” and direction, while diaphragm-guided crescendo-decrescendo indicators operationalize physiological feedback proportional to interval span. Together, these approaches tighten the perception–action loop that underwrites accurate intonation.

At the same time, the evidence base remains heterogeneous in measures, training dosage, and participant profiles, with limited longitudinal follow-up and cultural breadth. Future work should conduct adequately powered, longitudinal comparisons of practice schedules and scaffold-fading protocols; evaluate transfer to authentic performance settings; broaden cross-cultural repertoires; and integrate neurophysiological indices to track audio-motor coupling during learning.

Collectively, these findings situate interval-based solfege training as a model framework for studying the intersection of auditory cognition and instructional design. In sum, interval training functions as a multidimensional substrate for solfege pedagogy—linking perceptual encoding, tonal functionality, and embodied control. Programs that integrate relational listening with tonal context and embodied scaffolds are best positioned to yield durable, transferable gains in intonation accuracy and musical expressiveness, advancing both the psychology and pedagogy of musical learning.

## Data Availability

The original contributions presented in this study are included in this article/supplementary material, further inquiries can be directed to the corresponding author.
